# Diagnostic Efficacy of Cell Block Immunohistochemistry, Smear Cytology, and Liquid-Based Cytology in Endoscopic Ultrasound-Guided Fine-Needle Aspiration of Pancreatic Lesions: A Single-Institution Experience

**DOI:** 10.1371/journal.pone.0108762

**Published:** 2014-09-26

**Authors:** Shan-yu Qin, You Zhou, Ping Li, Hai-xing Jiang

**Affiliations:** 1 Department of Gastroenterology, the First Affiliated Hospital of Guangxi Medical University, Nanning, Guangxi, PR China; 2 Minerva Foundation Institute for Medical Research, Helsinki, Finland; 3 Department of Pathology, the First Affiliated Hospital of Guangxi Medical University, Nanning, Guangxi, PR China; University of Utah School of Medicine, United States of America

## Abstract

**Background:**

The diagnostic efficiency of endoscopic ultrasound-guided fine-needle aspiration (EUS-FNA) cytology varies widely depending on the treatment method of the specimens. The present study aimed to evaluate the diagnostic efficacy of cell block (CB) immunohistochemistry, smear cytology (SC), and liquid-based cytology (LBC) in patients with pancreatic lesions without consulting an on-site cytopathologist.

**Methods:**

This study prospectively enrolled 72 patients with pancreatic lesions. The EUS-FNA specimens were examined by SC, LBC, and CB immunohistochemistry. The diagnostic efficacy of the 3 methods was then compared. Patients’ final diagnosis was confirmed by surgical resection specimens, diagnostic imaging, and clinical follow-up.

**Results:**

Our results included 60 malignant and 12 benign pancreatic lesions. The diagnostic sensitivity (90%), negative predictive value (66.7%), and accuracy (91.7%) of CB immunohistochemistry were significantly higher than those of SC (70.0%, 30.0%, and 75.0%, respectively) and LBC (73.3%, 31.6%, and 77.8%, respectively) (all *P*<0.05). The combination of CB and SC, or CB and LBC, did not significantly increase the efficacy compared to CB immunohistochemistry alone.

**Conclusion:**

Our findings suggest that in the absence of an on-site cytopathologist, CB immunohistochemistry on EUS-FNA specimens offers a higher diagnostic efficacy in patients with pancreatic lesions than does SC and LBC.

## Introduction

Endoscopic ultrasound-guided fine-needle aspiration (EUS-FNA) cytology is widely used in the histological diagnosis of abdominal tumors, especially pancreatic lesions [1], [2]. Its diagnostic efficacy varies largely depending on a number of factors, including sample treatment, lesion characteristics, and cytopathologists’ expertise. Smear cytology (SC), liquid-based cytology (LBC), and cell block (CB) preparation are commonly used techniques for the analysis of specimens collected from EUS-FNA. SC, a traditional and standard method for cytological diagnosis, must be performed by an experienced cytopathologist in order to ensure the results’ accuracy [3], [4]. On the other hand, LBC, a thin-layer slide preparation technology, was developed to overcome the drawbacks of SC such as cell crowding and blood contamination [5]. CB provides more pathological information, when combined with histological examination such as hematoxylin and eosin (H&E) staining and immunostaining of serial sections compared with each method alone. Several studies have reported that CB improves diagnostic efficacy, and the implementation of immunocytochemistry is remarkably useful for the discovery of non-morphological markers [6], [7]. However, very few studies have evaluated and summarized the performance of these 3 methods in the examination of EUS-FNA samples. In the present prospective study, we assessed and compared the diagnostic efficacy of SC, LBC, and CB immunohistochemistry in specimens collected by EUS-FNA from patients with pancreatic lesions.

## Materials and Methods

### Patients and procedures

This study was prospectively conducted at the First Affiliated Hospital of Guangxi Medical University from January 2011 to January 2014. The nature and potential risks of the study were explained to all subjects before their written informed consent was obtained. Patients with coagulopathy according to medical history or laboratory coagulation tests were excluded. Consecutive patients with pancreatic lesions who underwent EUS-FNA performed by an experienced endoscopist (S.Y. Qin, who had 8 years’ experience with EUS-FNA) were included. Patients’ medical records were reviewed, and clinical information was extracted. The study was approved by the Institutional Ethics Review Board of Guangxi Medical University and conducted in accordance with the Declaration of Helsinki.

### EUS-FNA procedures

A linear array echoendoscope (Olympus Ltd., Tokyo, Japan) was used for EUS, and 22-gauge aspiration needles (Wilson-Cook Medical, Inc., Winston-Salem, NC, USA) were used for FNA. A transduodenal approach was employed for lesions in the head of the pancreas, whereas a transgastric approach was used for those in the pancreatic body or tail. Fine-needle tissue specimens were obtained under endoscopic ultrasound guidance. The needle was inserted into the lesion to its entire length to avoid contaminants of gastric or duodenal mucosa before aspirating. After the needle was retracted into the catheter, the entire catheter was withdrawn. Pre-designated passes were followed for each sample type: specimens from the first pass were used for SC, and those from the second and third passes were used for LBC and CB, respectively. On the basis of our preliminary results, the amount of sample procured from a single pass was usually enough for each method. However, that was not the case in 3 patients, who were subsequently excluded from the final analysis. In total, samples from 72 patients were examined by each technique. Aspirated materials were spread onto the center of a glass slide and fixed by skillful cytotechnicians. Slides were then delivered to the laboratory as soon as the procedure was completed.

### Specimen preparation

For SC, 2 smears were prepared for each aspirate. One was air-dried for modified Diff-Quick staining, and the other was fixed in ethanol for Papanicolaou staining. For LBC, aspirates were directly expelled into a single vial containing a liquid-based fixation medium for ThinPrep (Cytyc Corporation, Boxborough, MA, USA) processing. All staining procedures were performed after slides were delivered to the laboratory. The sodium alginate CB method was used to prepare CBs for H&E and periodic acid Schiff–Alcian blue staining. Aspirate was obtained via a separate pass in each patient and fixed in 10% buffered neutral formalin. Thin sections (4 µm) were cut from paraffin-embedded cell blocks on the following day and stained with H&E. After specimens were incubated in 10 mmol/L citrate buffer (pH 6.0) at 120°C for 10 min, p53 was detected with a monoclonal antibody (ZSGB-BIO, Beijing, China; 1∶100) after incubation for 20 min, whereas Ki67 detection with a monoclonal antibody (Maxim, Fuzhou, China; 1∶100) was performed for 30 minutes at room temperature. The slides were stained in an automated immunostainer using a Dako Cytomation EnVision-HRP Detection Kit (Dako, Glostrup, Denmark). In the present study, H&E staining and immunostaining were performed for all specimens used for the CB method.

### Definitions

An experienced cytopathologist blinded to the diagnostic method used for each specimen assessed the slides. Patients’ final diagnosis was decided according to diagnostic imaging findings throughout the follow-up period and cytohistological analysis after surgical resections. The mean follow-up time was 12 months (range, 3–16 months). Cytological results from all methods were reported as follows: (1) definite malignancy, (2) suspicion of malignancy, (3) benign cytology, (4) inadequate for diagnosis. Patients were stratified to the “malignancy” group when their results were either (1) definite malignancy or (2) suspicion of malignancy [8].

### Statistical analysis

Categorical data were analyzed by the chi-square test and Fisher’s exact test as appropriate. Sensitivity, specificity, positive predictive values (PPV), negative predictive values (NPV), and accuracy were calculated in a 2×2 contingency table. A *P* value of <0.05 was considered statistically significant. Data were analyzed using the SPSS version 18.0 for Windows (SPSS, Inc., Chicago, IL, USA).

## Results

### Patient characteristics

The present study included 72 patients (58 men and 14 women). The mean age of all patients was 54.6 years (range, 24–70 years). The lesion sizes ranged from 0.8×0.9 cm to 9.6×9.8 cm. Fifty lesions were located in the head of the pancreas, whereas 22 were in either the body or tail of the pancreas. Three needle passes were made in each lesion on average. Fifty-one patients were diagnosed by cytohistological analysis of the surgical resected samples, whereas diagnoses were based on imaging and serology test results during the follow-up period in the remaining 21 patients. Sixty patients were diagnosed with a malignant tumor, including 54 pancreatic adenocarcinomas, 4 pancreatic neuroendocrine tumors (PET), and 2 solid pseudopapillary tumors of pancreas (SPTP). Benign pancreatic lesions were determined in 12 patients, including 2 cases of pancreatic tuberculosis and 10 cases of chronic pancreatitis.

### Diagnostic rates in pancreatic lesions

CB successfully detected 54 cases of malignant pancreatic lesions, which was higher than the detection rate of SC and LBC. However, all 3 methods were able to correctly determine 12 cases of benign lesions (Table 1).

**Table 1:** Results of SC, LBC, and CB tests in benign and malignant pancreatic lesions.

Final diagnosis	n	SC			LBC			CB		
		+	±	−	+	±	−	+	±	−
+	60	42	0	18	44	0	16	54	6	0
−	12	0	0	12	0	0	12	0	0	12

SC: smear cytology; LBC: liquid-based cytology; CB: cell block histology; +: malignant; ±: indeterminate; −: benign.

When the lesions were categorized into various types, CB immunohistochemistry successfully detected 48 of 54 pancreatic adenocarcinomas, which was slightly higher than the detection rates of SC and LBC. Moreover, while CB accurately determined all the positive cases of SPTP and PET, SC and LBC failed to detect SPTP and performed inadequately on PET, detecting only 2 of 4 cases. However, SC and LBC were able to detect 3 pancreatic adenocarcinoma cases, which were not detectable by CB. The detection rates of pancreatic tuberculosis and chronic pancreatitis were similar among these 3 methods (Table 2).

**Table 2:** Results of SC, LBC, and CB tests in pancreatic lesions of various types.

Lesion types	Final diagnosis	SC	LBC	CB
Adenocarcinoma	54	41	42	48
SPTP	2	0	0	2
PETs	4	1	2	4
Pancreatic Tuberculosis	10	10	10	10
Chronic pancreatitis	2	2	2	2
Total	72	54	56	66

SC: smear cytology; LBC: liquid-based cytology; CB: cell block histology; PETs: pancreatic neuroendocrine tumors; SPTP: solid pseudopapillary tumor of the pancreas.

### Diagnostic efficacy in pancreatic lesions

The sensitivity (90%), NPV (66.7%) and accuracy (91.7%) of CB immunohistochemistry were significantly higher than those of SC (70.0%, 31.6%, and 75.0%, respectively) and LBC (73.3%, 30.0%, and 77.8%, respectively) (*P*<0.05). The specificity and PPV were comparable among the 3 methods (Table 3).

**Table 3:** Diagnostic efficacy of SC, LBC, and CB methods in pancreatic lesions.

	SC	LBC	CB
Sensitivity, % (n)	70.0% (56.8%–81.2%)	73.3% (60.3%–83.9%)	90.0% (79.5%–96.2%)*
Specificity, % (n)	100% (73.5%–100%)	100% (73.5%–100%)	100% (73.5%–100%)
PPV, % (n)	100% (91.6%–100%)	100% (92%–100%)	100% (93.4%–100%)
NPV, % (n)	30.0% (22.7%–59.4%)	31.6% (24.5%–62.8%)	66.7% (41.0%–86.7%)*

SC: smear cytology; LBC: liquid-based cytology; CB: cell block histology; PPV: positive predictive value; NPV: negative predictive value.

*P<0.05 CB compared to SC and LBC.

### Diagnostic efficacy of different method combinations

Furthermore, we compared the diagnostic efficacy of CB performed in combination with SC or CB with LBC to CB alone. However, no significant differences in sensitivity or accuracy were observed between the method combinations and CB alone (Table 4).

**Table 4:** Comparison of diagnostic efficacy among SC, LBC, and CB methods.

	CB	SC+CB	LBC+CB
Sensitivity, % (n)	90.0% (79.5%–96.2%)*	91.7% (81.6%–97.2%)	93.3% (83.8%–98.2%)
Specificity, % (n)	100% (73.5%–100%)	100% (73.5%–100%)	100% (73.5%–100%)
PPV, % (n)	100% (93.4%–100%)	100% (93.5%–100%)	100% (93.6%–100%)
NPV, % (n)	66.7% (41.0%–86.7%)*	70.6% (44%–89.7%)	75.0% (47.6%–92.7%)

SC: smear cytology; LBC: liquid-based cytology; CB: cell block histology; PPV: positive predictive value; NPV: negative predictive value.

### Complications

No serious complications were observed in the present study.

## Discussion

EUS-FNA has been increasingly used for cytological and/or histological assessment of pancreatic lesions as a safe and cost-efficient method [9], [10], [11]. In the present study, EUS-FNA was performed in patients with pancreatic lesions using 22-gauge aspiration needles. The specimens were analyzed by SC, LBC, and CB immunohistochemistry. Our findings demonstrated that CB immunohistochemistry provided more reliable and accurate results than SC and LBC did in the diagnosis of pancreatic lesions, suggesting that CB might be a superior method for examining pancreatic samples.

SC is a well-established method for cytological diagnosis of tissue samples obtained by EUS-FNA. A number of studies have reported that the sensitivity, specificity, and accuracy of SC for the detection of solid pancreatic masses were 85–90%, 95–100%, and 85–95%, respectively [12], [13], [14], [15]. Despite its decent performance, SC has several limitations. For example, ethanol fixation of cells may remove Papanicolaou staining. In addition, Giemsa and Diff-Quick staining may cause the cells to swell and degenerate. Moreover, specimens for SC are easily contaminated with materials from the gastrointestinal tract, which would undermine diagnostic accuracy. Furthermore, SC in many cases is inadequate owing to sampling error. Such an issue could be easily overcome by applying the rapid on-site cytology evaluation (ROSE) technique. Iglesias-Garcia et al [16] have found that the presence of an on-site cytopathologist significantly decreases the number of inadequate samples and improves diagnostic sensitivity and overall accuracy. However, in many developing countries, the presence of an on-site cytopathologist for each patient is indeed difficult owing to financial constraints. In the present study, although we did not employ an on-site cytopathologist, the diagnostic accuracy and reliability of SC for the detection of pancreatic lesions were similar to those previously reported [17], [18]. We found that SC successfully detected all the benign pancreatic lesions in spite of the small sample size. Thus, future studies with a large number of subjects are warranted to confirm our findings.

LBC has overcome the drawbacks of SC, such as cell crowding and blood contamination, by using a single layer of cells [19]. The technique is widely employed for the diagnosis of uterine cervical cancer, bile duct cancer, and gall-bladder cancer with high accuracy [20], [21]. Some studies have compared the diagnostic performance between LBC and SC for a variety of diseases. However, their results were controversial. Son et al [22] found that LBC could reveal more cellularity with a cleaner background and better cytomorphologic features, and that the diagnostic sensitivity of LBC was remarkably higher than that of SC. In contrast, Sykes et al [23] reported similar sensitivity by LBC and SC for the detection of cervical intraepithelial neoplasia 2/3. To date, few studies have compared the diagnostic efficacy between LBC and SC performed on EUS-FNA specimens from patients with pancreatic lesions. LeBlanc et al [24] reported that LBC was inferior to SC for the detection of pancreatic malignancy in a study that employed an on-site cytopathologist during the EUS-FNA procedure. In addition, a prospective study found that LBC was less accurate than SC after EUS-FNA in patients with pancreatic malignancy [8]. We found that the diagnostic efficacy of LBC was relatively higher than that of SC. However, the difference was not statistically significant. The rates of successfully detecting malignant pancreatic lesions were similar between these 2 methods. Therefore, when cost and technical details are considered, it is hard to conclude that LBC is a better alternative to SC.

CB has been recognized as a powerful technique for evaluating tissue architecture and determining its histological features. Moreover, immunostaining of serial sections is commonly used for a definitive cytohistological diagnosis [25], [26]. Compared to SC and LBC, CB immunohistochemistry was much more reliable owing to the gold standard of histological staining regardless of lesion location or approaching routes [27], [28]. A prospective study reported that the efficacy of CB immunohistochemistry was significantly higher than that of SC for the detection of malignancies and benign lesions [18]. Ardengh et al [29] found that CB immunohistochemistry provided a higher sensitivity and accuracy than SC did in detecting pancreatic tumors from EUS-FNA specimens of 611 patients. In addition, Sai et al [30] reported that the sensitivity, specificity, PPV, and NPV of CB were 92%, 100%, 100%, and 97%, respectively for discriminating benign and malignant branch-duct type neoplasms. Raddaoui et al [28] showed that CB had a 74% sensitivity, 100% specificity, 100% PPV, and 76.9% NPV for the detection of pancreatic lesions.

In the present study, the sensitivity and specificity of CB immunohistochemistry were similar to those reported by Noda et al [18], whereas our sensitivity was higher than the values published by Ardengh et al [29], Haba et al [31], and Kopelman et al [27] (Table 5). Such differences might be the result of heterogeneous sample sizes and types, such as solid versus cystic pancreatic lesions. Moreover, we found that CB immunohistochemistry had a significantly higher efficacy than that of SC, which was in agreement with the findings by Noda et al [18], Ardengh et al [29], Haba et al [31], and Kopelman et al [27]. Additionally, CB was able to detect almost all malignant pancreatic lesions in this study, especially SPTP and PETs, which was significant to treatment guidance. Unlike the report by Noda et al [18], H&E staining and immunostaining were performed on all specimens used for the CB method in this study. We found that both staining procedures exerted similar diagnostic performance. Immunostaining did not show any significant contribution to the CB diagnosis over and above H&E histology.

**Table 5:** Comparison between CB and SC methods for pancreatic lesion diagnosis via endoscopic ultrasound-guided fine-needle aspiration.

	n	CB				n	SC			
		Sensitivity	Specificity	PPV	NPV		Sensitivity	Specificity	PPV	NPV
Noda et al	85	92.0%	100%	100%	88.9%	85	60%	62.5%	93.8%	60.6%
Ardengh et al	178	85.2%	93.1%	98.4%	55.1%	178	61%	100%	100%	36%
Haba et al	956	74.9%	78.6%	99.8%	38.2%	983	88.0%	95.2%	100%	54.5%
Kopelman et al	85	73.0%	94.0%	96.0%	66.0%	99	63.0%	100%	100%	63.0%

SC: smear cytology; CB: cell block histology; PPV: positive predictive value; NPV: negative predictive value.

The combination of CB and SC or LBC did not significantly increase the diagnostic efficacy compared to CB alone, indicating the relatively accurate detection rate of CB compared with SC or LBC alone. In contrast, 2 cases of pancreatic adenocarcinoma were detected by LBC and 1 by SC while CB failed, suggesting that LBC and SC might be superior in some specific cases. Furthermore, CB typically has a longer specimen turn-around time owing to complicated sample processing and immunostaining procedures. For CB, the case results would not be available until at least the following day because of overnight tissue processing. This major drawback of CB remarkably increases the cost compared to SC and LBC methods [32]. Furthermore, several issues are commonly observed with cell immunostaining, such as cell loss, disruption of cells and leakage of antigen, and high background staining owing to blood and necrotic materials. These limitations of CB should be considered cautiously prior to its implementation. Therefore, when selecting an appropriate diagnostic method, physicians should take not only its efficacy but also financial and logistic costs to accommodate each patient’s situation into account.

Our study has several limitations. First, pre-designated passes were conducted for each sample type, which might present a lack of standardization and cause sampling errors. In addition, selection bias might be inevitable and thus negatively affect the robustness of our results. Therefore, it should be interpreted with caution. Second, not all patients’ lesions were confirmed by histological examination of the resected specimens. Third, the sample size of the present study was relatively small. Finally, no on-site cytopathologist was employed for SC screening. Thus, the impact of ROSE on the performance of SC and CB was not evaluated. Larger sample size studies in combination with ROSE are warranted to assess the diagnostic accuracy and reliability of CB, SC, and LBC tests.

In conclusion, our results suggest that CB immunohistochemistry after EUS-FNA might offer a higher diagnostic efficacy than SC without ROSE and LBC in patients with pancreatic lesions.
